# Determining factors of participation and success rates in research funding competitions: Case study

**DOI:** 10.1371/journal.pone.0272292

**Published:** 2022-07-29

**Authors:** Valentina Diana Rusu, Mihaela Mocanu, Anca-Diana Bibiri

**Affiliations:** Department of Social Sciences and Humanities, Institute of Interdisciplinary Research, ‘Alexandru Ioan Cuza‘ University of Iași, Iași, Romania; Public Library of Science, UNITED KINGDOM

## Abstract

Research and innovation play a key role in generating smart and sustainable economic growth. By producing new knowledge, the research contributes to the development of new and innovative products, processes, and services, which in turn lead to increased productivity, industrial competitiveness, and, ultimately, the prosperity of the community as a whole. However, all research, development and innovation activities depend on the financial resources made available, as specific financing accelerates the production and dissemination of the best ideas and practices, as well as their role in meeting the challenges our society deals with nowadays. Our study aims to identify the determining factors for the researcher’s participation and success rates in research funding competitions. The goal of the research is to understand how variables such as age, gender, main field, affiliation, and scientific rank can affect the access to funding opportunities available for research and innovation. The study relies on a questionnaire-based survey conducted with 243 early-career and senior researchers from many state universities across Romania. For an in-depth analysis of the factors that influence the success rate in research competitions, in the present approach, we used both graphical and econometric methods. A binary logistic regression modelling was performed in order to explain the relationships between variables. Among other considerations, our findings revealed that in all main research fields, scientific rank and gender are important features for raising the participation and success rate in research funding competitions.

## Introduction

The public system for research and innovation (which includes the upper education institutions and other public organizations that conduct research and innovation activities) plays a key role in creating and stimulating the knowledge required by innovating enterprises, allowing them to consolidate their efforts targeted at research and innovation. The quality of the public system for research and innovation is assessed based on relevant performance indicators, such as scientometrics (which measures the impact of scholarly publications on the production of new knowledge), the number of grants/funded research projects won by the researchers from a certain country and the number of universities included in international rankings for high performance in research. A comparative analysis of the quality of the public RandI system in the EU member states indicates a relatively lower quality in Eastern European countries compared to other member states. Moreover, the analysis points to a less significant discrepancy between the Northern and Southern areas, since Greece, Portugal, Spain, Cyprus, Malta and Italy are slightly below the EU average and have an intermediate position between Eastern and Northern European countries. To a large extent, these differences derive from lower public investment in research and development in the countries ranked in the lower positions.

The study of Zacharewicz et al. [1] also shows that there are major variations in public research funding allocation systems in Europe. The authors grouped the countries analysed into two clusters and noted that within both of them there are large variations in the methodologies adopted, the evaluation criteria and also in other metrics taken into account for evaluation.

According to the data provided by the Statistical Office for the European Union (EUROSTAT), in 2019, the highest percentage of the GDP allocated to research and development expenditures, of over 3%, was registered in Sweden (3.3%), Austria (3.1%) and Germany (3.1%). These three countries are closely followed by Denmark (2.9%), Belgium (2.8%) and Finland (2.7%), with values close to 3% of the GDP. On the other hand, eight member states allocated less than 1% of the GDP to research and development expenditures: Romania (0.4%), Malta (0.6%), Cyprus (0.6%), Latvia (0.6%), Ireland (0.7%), Slovakia (0.8%), Bulgaria (0,8%) and Lithuania (0.9%). Eurostat data indicates that in the past decade, the GDP percentage allocated to research and development expenditures has increased in 19 of the member states, with Belgium occupying the top position (with an increase of 0.8 percentage points), Poland (0.6 percentage points), the Czech Republic (0.6 percentage points) and Greece (0.6 percentage points), whereas in six of the member states this GDP percentage decreased, the most significant decline being recorded in Finland (minus 0.9 percentage points) and Ireland (minus 0.8 percentage points). The GDP percentage allocated to research and development expenditures remained stable in France and Sweden.

Equality between women and men is one of the EU’s founding values, enshrined in the European Treaties. Since 2012, ‘gender equality and gender mainstreaming in research’ has been one of the priorities in achieving the European Research Area (ERA). At European level, the funding success rate was higher for men than women by 3.9%, showing that gender differences persist in access to funding. Among the EU-27 Member States and Associated Countries, this funding difference in favour of men was seen in most countries with available data (19 of 28), with the largest difference found in Slovakia (7.7%). Conversely, in nine EU-27 Member States and Associated Countries (BE, BG, DK, LV, LU, MT, RO, SI, IS), the funding success rate was higher for women than men. Iceland had the largest difference in favour of women (10.6%), followed by Bulgaria (7.8%). Funding success rates were closer to gender parity (difference of -0.5 to 0.5%) for Germany (-0.2), Slovenia (0.4), Finland (0.0) and Sweden (-0.1).

At European level, in all fields of RandD except Agricultural Sciences and Humanities and Arts, women were less successful than men when applying for research funds. More specifically, the largest difference in favour of women was in Agricultural Sciences (0.8), while the largest difference in favour of men was in Natural Sciences (-2.5). There was some variation at country level. The difference in funding success rate was in favour of women in eight of the EU-27 Member States and Associated Countries in Natural Sciences (BG, DK, LU, NL, RO, FI UK, NO), Medical Sciences (BG, DK, DE, IT, HU, RO, SI, IS), Agricultural Sciences (DK, EE, LV, HU, AT, RO, SE, TR) and Humanities and Arts (DK, EE, NL, AT, PL, SI, FI, NO). In more than half of the countries with available data Engineering and Technology (BG, DK, DE, LV, HU, AT, PT, RO, FI, SE, IS, NO, TR) and Social Sciences (BG, DK, EE, CY, LV, RO, SI, SE, UK, IS, CH, TR, IL), the difference in funding success rate was in favour of women.

The public research and innovation (RandI) system in the EU Member States generally relies on two broad categories of financial resources: (a) institutional funding, defined as a direct and global financial flow directed at public research such as institutes, academies or universities. The criteria and manner in which the amounts are distributed vary from one country to the other, capitalizing on specific algorithms for the assessment of the outcomes; (b) project-based funding, defined as the allocation of funding based on an open and competitive selection process for an entity carrying out the research activity proper (i.e, researcher, research group, research centre, network of researchers). By the nature of the activities undertaken through the research project and the funding contract, the research activity carried out within the research projects is limited in terms of coverage, budget and time frame. Responsible intermediate agencies have been set up at national level in order to manage the project competitions, such as Agence Nationale de la Recherché – ANR in France, Deutsche Forschungsgemeinschaft–DFG in Germany, Executive Agency for Higher Education, Research, Development and Innovation Funding–UEFISCDI in Romania.

Starting from the principle that funding is essential for conducting research activities and that each researcher is directly interested in attracting financial resources by participating in competitions for research projects, our research focused, on the one hand, on establishing the participation rate of academic research staff in such competitions and, on the other hand, on identifying the factors that influence the success rate of those applicants. The case study, based on the situation of researchers in Romania, was supported by quantitative data, collected following the online administration of a questionnaire dedicated to Romanian academic research staff from universities and state academic institutes. The data collected through the online survey was compared to statistical information regarding the participation/success rate of researchers in competitions organized by the Executive Agency for Higher Education, Research, Development and Innovation Funding (UEFISCDI). As a public institution subordinated to the Ministry of Education and Research, UEFISCDI supports studies aimed at substantiating the distribution of state funds to universities, as well as the administrative coordination of some programmes and sub-programmes included in the National Plan for Research, Development and Innovation. As a research funding agency and similarly to other international agencies, UEFISCDI organizes competitions for research projects and monitors the implementation of projects accepted for funding, managing approximately 22% of the public funds allocated to research, development and innovation in our country.

The case study focusing on the Romanian research public system relates to national strategic documents regarding the financing of research, development and innovation, as well as the instruments specifically conceived as a form of support for academic staff engaged in research activities. The value-added of our study derives, first and foremost, from the fact that it identifies and ranks the factors that influence the participation and success rate of the researchers in funding competitions. Our study fills a gap in the literature by analysing the relationships between participation and success rate and variables such as research field, career stage and gender. We also correlated the research results with the statistical data provided by the financing authorities. The research outcomes can be capitalized by decision-makers and authorities in order to develop optimal financial mechanisms and instruments to stimulate participation rate in research competitions, leading to better public policies in the field.

The paper is structured as follows: the first section presents a literature review related to our topic research; the following section contains the methodology, describing data collection methods, the sample, and the research instrument used; in the third section, which presents the results, we discuss and analyse the factors that influence the participation and success rate in research funding competitions; the last section summarizes the conclusions of our research.

## Literature review

Funding has been viewed in the literature as one of the main determinants of scientific activities. The benefits of competition in terms of knowledge production have been emphasized [2] and the impact on the researcher’s individual productivity was also highlighted [3]. Securing funding is one of the most important factors for a researcher, enabling him to carry out research projects. Researchers showed that taking part in a grant competition has positive effects on the scientific productivity, learning, and collaboration of scientists. The study of Ayoubi et al. [3] focused on the utility for researchers to spend time writing proposals to raise money for their research. The findings of their study show that there are benefits from the time spent writing proposals, even if they do not earn funding for the project. The authors claim that simple participation has benefits. Researchers taking part in a research grant competition boost their number of publications and average impact factor while extending their knowledge base and their collaboration network regardless of the result of the competition [3]. The ability to raise funds is becoming a key skill in managing research laboratories [4] and a base in the evaluation of scientists’ performances along with publication records.

The current dominant mechanism for allocating public funding to research projects is grant peer review, although the idea of random grant allocation is also being discussed as an alternative to peer review [5]. Funding for research is allocated through a competitive bidding process: academics write grant proposals, and then the proposals are reviewed for quality, and a panel of experts decides which proposals will be funded. However, researchers spend an increasing number of hours writing grant proposals with uncertain outcomes. Not everyone is successful in obtaining the necessary funds. What are the ingredients that ensure success in research funding competitions? and What are the influencing factors that positively affect the participation and success rates in such research funding competitions?

The results obtained in the literature are varied. Some studies focused on the role of the quality of the proposal, previous publications and other factors in this field on the success rate in funding competitions [6, 7]. Other studies have shown that not only the quality of the proposal is significant for ensuring success in a funding competition. The frequent success amongst those who obtain grants is usually associated with a number of factors that may be viewed as conferring advantages. For example, the study of Viner et al. [8] pointed out that in the award of the research grants there exist ethnicity and gender biases. In another study, Lawson et al. [9] showed that individual competitive funding is linked to several individual characteristics such as: career stage, prior performance, gender, and socio-political capital. The authors also examined the impact of caring for a young child, and they found that women produce lower impact research (motherhood penalty). Their findings confirm the Matthew effect that is an important driver for funding success, as researchers who previously obtained funding for their projects are more likely to succeed again, producing increasing distinction [9–11]. This advantage will lead to a more successful careerbecause it offers the chance to publish more, to get more citations, awards and employment records. This aspect is very important especially in the early-career stage because it motivates researchers to compete for research funding again (while grant rejection has a negative consequence on future participation in funding contests). On the other hand, the Matilda effect underlies the idea that women’s achievements do not receive the same recognition as men, gaining fewer awards and prizes in research [12, 13].

Demographic variables such as age, gender, main field, affiliation, and scientific rank have a determinant role in obtaining research funding. We discuss in the following the influence of those variables on the participation process and in the success rate. A significant role is played by the research field where there are differences concerning the parity of men and women in applying and also getting grant allocation. There are certain disciplines (such as the social sciences) where equality is better upheld given that women have a more equal role to men [14]. As van der Lee and Ellemers consider, gender disparities were most pronounced in scientific disciplines in which female applicants were more visibly present and larger numbers of applications had to be processed (i.e., life sciences and social sciences) [15]. For example, in Iceland [16], the gender distribution is generally equal: women and men globally show comparable success in their granted amounts and numbers, particularly in the social sciences, whereas women are more likely to receive higher grants than men in female-dominated fields like education, and men are substantially more likely to be awarded grants and receive higher amounts of funding in male-dominated fields such as engineering, natural sciences, health sciences, and humanities. The relative absence of women in the STEM fields thus produces a tenuity of female role models and networks, where women are demanded to comply with the discipline’s masculine culture to advance into their careers [17]. In this sense, Casad et al. [18] consider that there are three factors that contribute to the gender inequalities in STEM: numeric underrepresentation and stereotypes, lack of supportive social networks, and chilly academic climates, while proposing solutions to this state: recruiting diverse applicants (e.g., training search committees), mentoring, networking, and professional development; and improving academic climate.

Widespread in scientific communities, gender effects in research funding show various intensities across countries, disciplines and organisational levels. Most studies have shown that women’s likelihood of receiving research funding is lower than that of their male colleagues. The research on this gender gap in academia has focused on post-PhD academics, making it difficult to discern whether the female disadvantages in number of publications, previous grants, maternity leave, and *h*-indexes are at the root of the gender gap in received funding, or whether it is due to a more fundamental gender bias in academia. A great body of literature admits the idea that there are still disparities in the treatment of women and men in the grant funding process, issues central to understanding differences in female and male career trajectories [19]. However, there are more factors involved in developing professional status in research: structural biases related to how academic scientific institutions function [20], gender biases [21], and differences in field, career, stage, or scientific productivity [22].

Another issue is the idea that women apply less [15, 23, 24], and as a consequence, they succeed less often [15, 16, 25–27]. Deriving from here is the fact that this could be one of the key causes for women having less successful scientific careers [28].

Representing another determining factor in funding research, the career stage is a potential source of bias in grant awards. Early-career female applicants for research funding have been found comparable to men competitors in submission, success rate and grant amounts. On the contrary, as women advance in their careers, they receive fewer grants [14]. According to the years elapsed from the doctoral degree, there are different stages of research activities across countries, regions and research systems. For example, in Romania, there are specific financing tools that concern young researchers (Postdoctoral research projects: PD and Research projects to stimulate young independent teams: TE) and experienced researchers (Exploratory research projects: PCE and Complex Border Research Projects: PCCF).

To summarize, academic excellence such as employment, performance evaluation, the grade of payment, and attainment of research grants represent a more challenging reality for women researchers than for male researchers, and visible progress toward gender equality especially in STEM fields is slow.

## Methodology

The empirical analysis from this study is focused on a sample of teachers and researchers from Public Romanian Universities and Research Institutes. Given the fact that university teachers also carry out research activities, in the following, we will include the two categories under the umbrella of researchers. In order to carry out the quantitative analysis, we developed a questionnaire entitled *Participation and success in Research Funding Competitions*. The items from the questionnaire focus on identifying the experiences of researchers in participating in research project funding competitions. The questionnaire comprises a set of 17 items. The time required to complete it is around 10 minutes. Even if our study involved the participation of individuals, they were not required to give written or oral consent because they were not asked for personal identification data, were informed of data protection and the data obtained were processed anonymously. Regarding ethics, this paper was carefully anonymized in order not to disrupt respondents’ privacy and not to harm them or other individuals involved. This paper was written in the spirit of transparency, specifically concerning the methodology and the evaluation of research weaknesses.

The data for the study was collected by an online questionnaire survey, during the period July-October 2021. The questionnaire comprises two types of questions: some questions where the answers are formulated according to the Likert scale of 5 points (1- total disagreement; 2- partial disagreement, 3- neutral, 4- partial agreement, 5- total agreement), other questions with only one allowed answer, but also with several possible answers. The questionnaire ends with some socio-demographic questions.

In the initial phase, we made a qualitative and quantitative pre-test, on a small number of respondents (10 people) in order to verify the understanding of the content but also to make changes so that the questions are as clear as possible and follow our main objective. Starting from the recommendations received in the pre-test phase, we have adjusted the questionnaire and then we applied it at an extended level. The total number of valid answers we received following the online application of the questionnaire is 243. The structure of our sample is presented in Table 1 below.

**Table 1:** The structure of the sample.

	Number	Percentage
***Home university***		
UAIC	124	51.0%
University of Craiova	36	14.8%
Dunărea de Jos University of Galați	23	9.5%
Other	60	24.7%
*Total*	*243*	*100%*
***Gender***		
Female	99	40.7%
Male	135	55.6%
Did not want to say	9	3.7%
*Total*	*243*	*100%*

Source: authors own calculations

Therefore, the sample is formed 51% from respondents from”Alexandru Ioan Cuza” University of Iași (UAIC), 15% from University of Craiova, 9% from Dunărea de Jos University of Galați. The remaining 25% of the respondents came from University of Bucharest, University of Medicine and Pharmacy Iași, Romanian Academy Iași, Gheorghe Asachi Technical University of Iasi (TUIASI), University of Petroșani, West University of Timișoara, Iași University of Life Sciences, Technical University Cluj-Napoca,”Ștefan cel Mare” University of Suceava and Romanian Academy Bucharest.

The gender distribution shows that 55% of the respondents were males and 41% females. Around 4% preferred not to say their gender.

Based on this sample, we aimed at analysing the factors that determine the participation and the success rate of the Romanian researchers in competitions for financing research projects. Starting from the findings obtained by other studies in the literature mentioned in the previous section but also from the main objective of this paper we have formulated a set of hypotheses that are the base of the quantitative analyses performed in this study, as follows:

*Hypothesis 1*: *The field of research has a significant influence on the participation and success rate of researchers in research funding competitions*.
*Hypothesis 2*: *The professional level experience has a significant influence on the participation and success rate of researchers in research funding competitions*.
*Hypothesis 3*: *The applicant gender has a significant influence on the participation and success rate in research funding competitions*.

To test the formulated hypotheses, we used a series of quantitative methods, such as: graphical method, to present in a clearer way the results and to facilitate a rapid assimilation and understanding of information; and the method of comparison. Also for a more in depth analysis in identifying the factors that influence the participation and success rates of researchers in research projects funding competitions we used binary logistic regression modelling. The equations for the logit models applied are:

Model1:Participation=β0+β1⋅Mainfield+β2⋅Specificfield+β3⋅AcademicRank+β4⋅Gender+β5⋅Age+m (1)

Model2:Success=β0+β1⋅Mainfield+β2⋅Specificfield+β3⋅AcademicRank+β4⋅Gender+β5⋅Age+m (2)

Where *βi* represents the coefficients and *m* is the error term.

We use two dependent variables. The first variable expresses the *rate of participation to research projects funding competitions*, and takes the value 1 for the respondents that said that they participated in at least one competition in the last five years and the value 0 for those who did not participate in any competition in the last five years. The second dependent variable expresses the *success rate of the applicants*, and takes the value 1 for the respondents that said that they obtained financing at least for one project submitted in research funding competitions in the last five years and the value 0 for those who did not obtain financing.

The independent variables we are referring to are: *the age of the researcher*, *the main field of interest*, *the specific field of interest*, *the didactic or research rank* and *gender of the respondent*. For statistical data processing we used the SPSS software package. Thus, in order to perform the binary logistic regression modelling we quantified the independent variables mentioned so that they can be run by the program.

## Results and discussions

**Fig 1:**
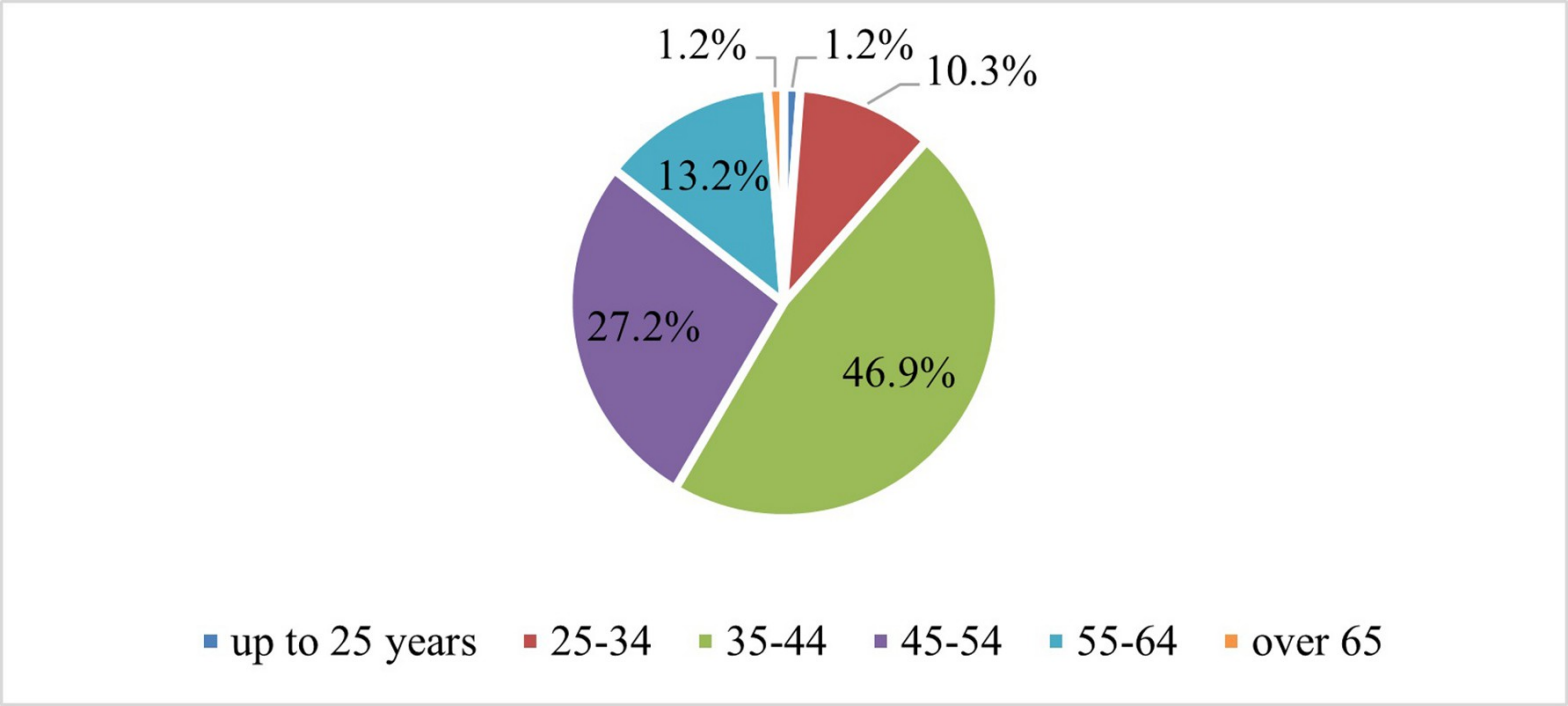
Sample structure according to the age of the respondents. Source: authors own calculations.

The results obtained from the data processing after the application of the questionnaire are detailed in this section. Thus, depending on age, almost half of the respondents are included in the age group between 35 and 44 years, and a little over a quarter in the age group 45–54 years. 13% of respondents are between 55 and 64 years old and 10% between 25 and 34. The extreme values of age, those who are up to 25 years old or those over 65 years old have low shares, both of 1%. This is justified by the fact that those up to 25 years old are usually still in the development phase of doctoral studies, not being employed as a researcher or university teacher. And, 65 being the retirement age, there are only a few persons who are still researching/applying for funds after this age, as we can notice in Fig 1.

**Fig 2:**
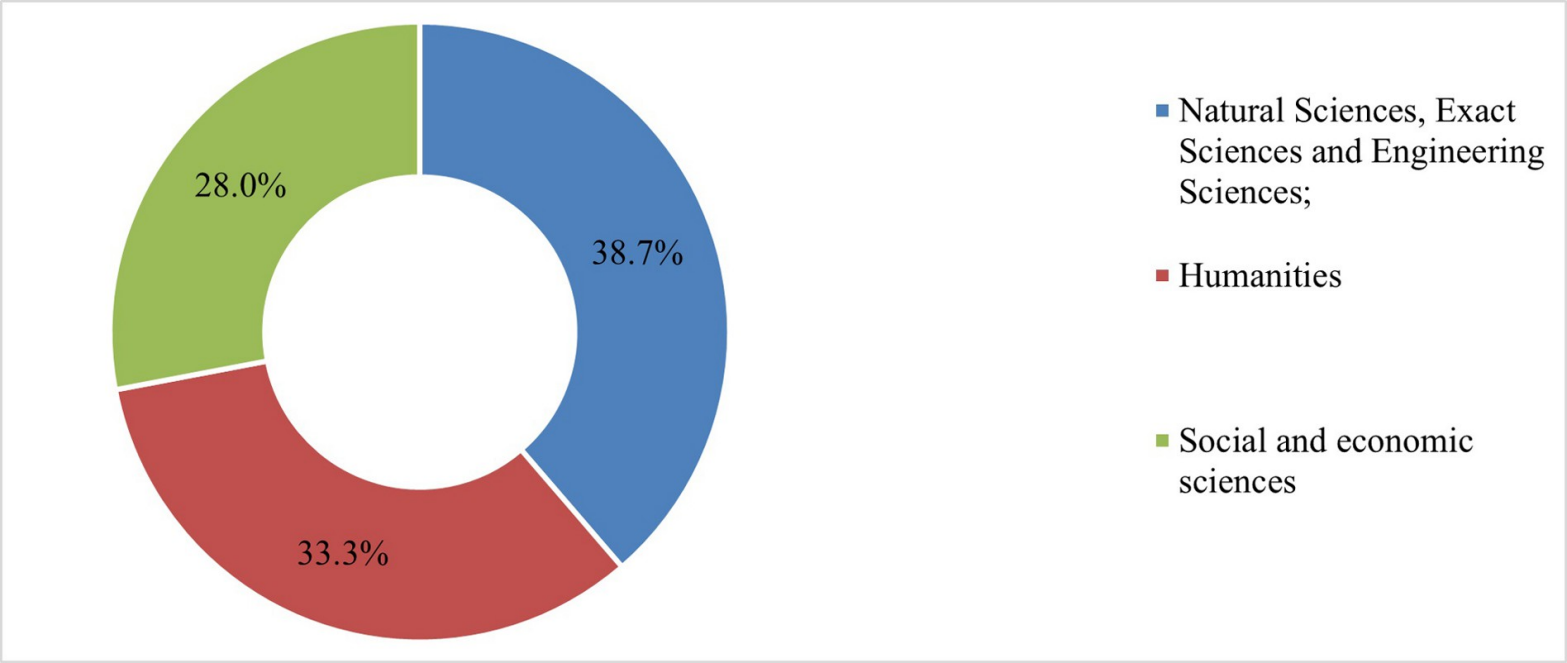
Fundamental areas of interest of the respondents. Source: authors own calculations.

In an analysis of fundamental areas of research, we notice (Fig 2) that the respondents are divided more or less evenly. Thus, for 38% of the respondents the field of interest falls into Natural Sciences, Exact Sciences and Engineering Sciences, for 33% the field of interest is Humanities, and for around 28% Social and Economic Sciences.

**Fig 3:**
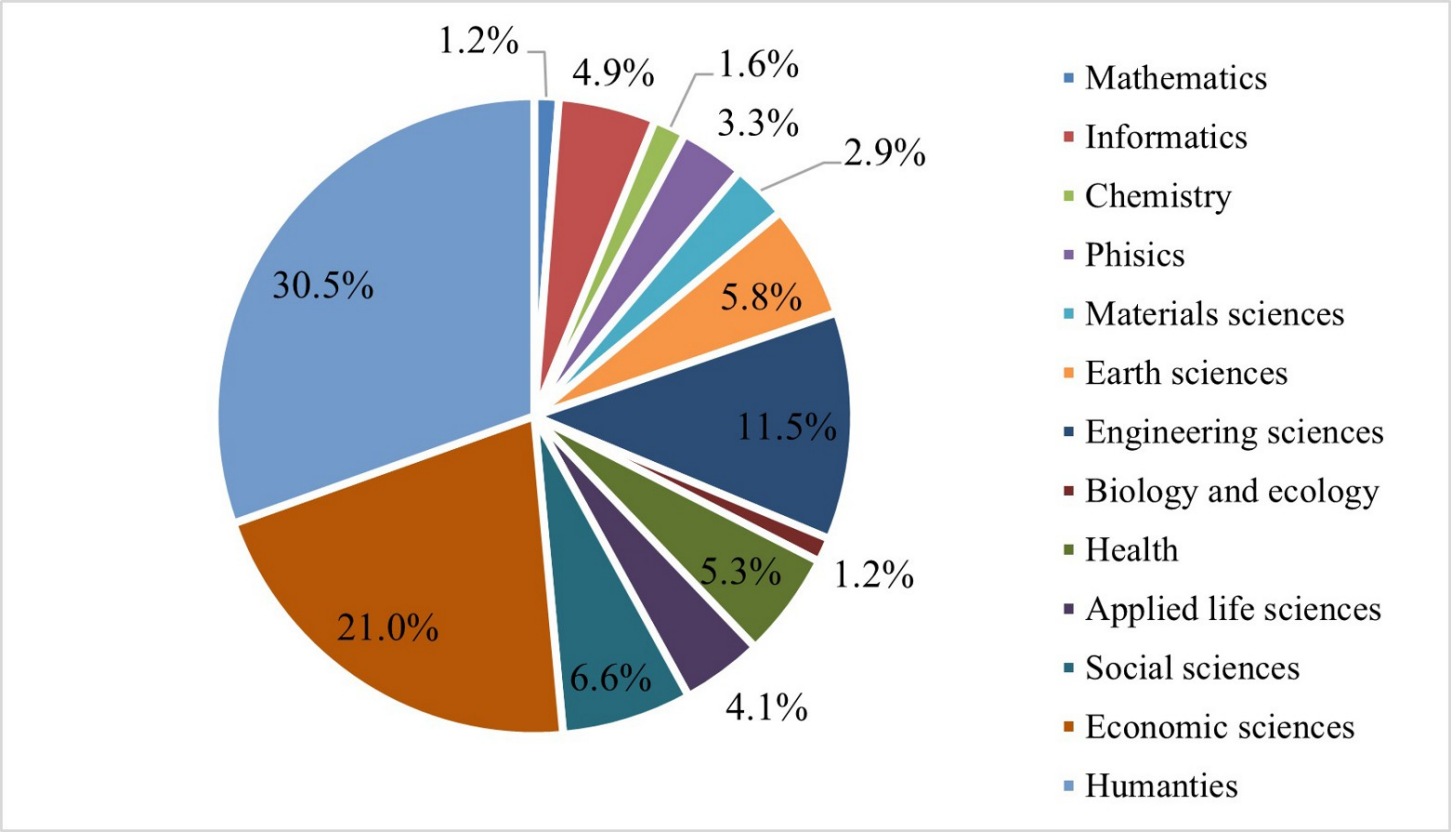
Specific areas of interest of the respondents. Source: authors own calculations.

Fig 3 shows the specific areas of interest of the respondents. The largest shares are held by the humanities and social sciences, which together hold about 50% of the interests of those who responded to the questionnaire. The remaining 40% is divided between the other fields, with higher shares for Engineering sciences (11%), Social sciences (approximately 7%), Health (5%) and Informatics (around 5%). The other specific areas of interest have shares of 4% or less.

**Fig 4:**
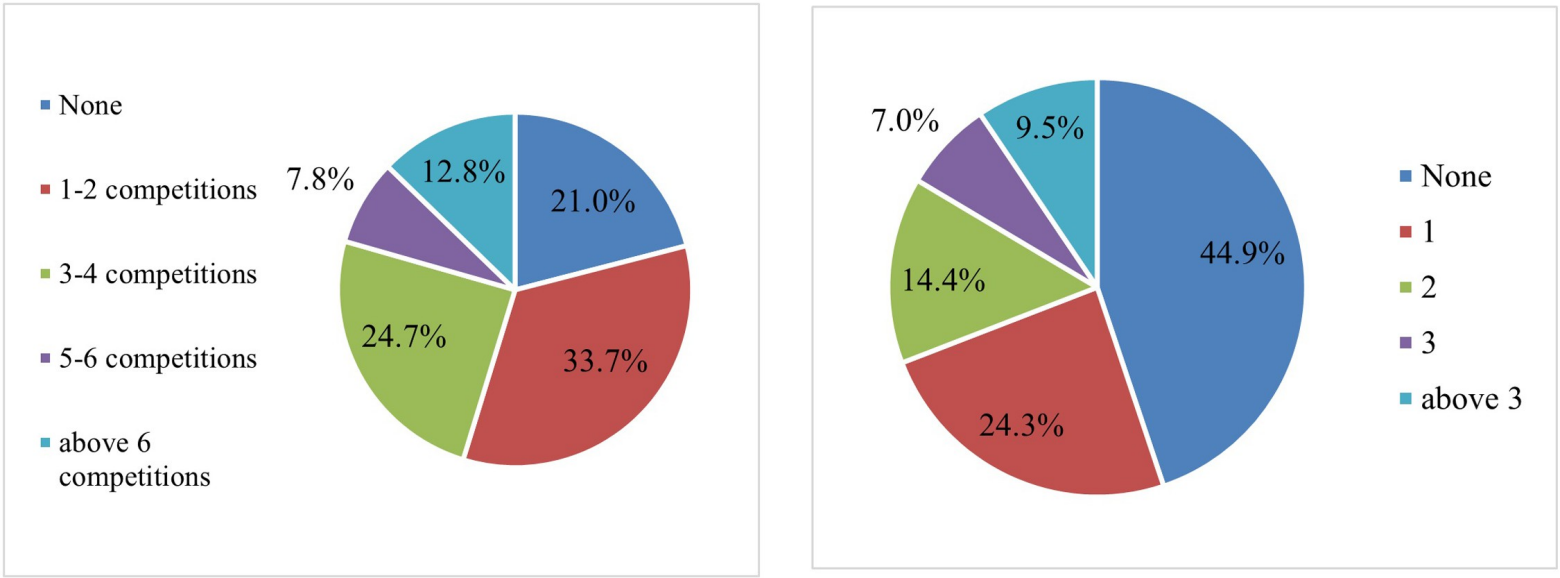
Number of competitions in which respondents participated in the last 5 years and number of applications that obtained funding. Source: authors own calculations.

Regarding the experience of the respondents related to the project submissions in previous research funding competitions, we took into account how many competitions the respondents applied in the last 5 years and how many of the applications received funding. The results are shown in Fig 4:

Thus, we note that the largest share of respondents, around 55%, participated in between one and four research projects funding competitions. Almost 21% did not participate in any competition. This result is to some extent worrying because it is a rather high share of those who did not participate in funding competitions at all. 20% of respondents participated in over 5 competitions in the last five years.

From those who applied to these competitions only about 55% obtained funding. 24% obtained funding for only one project application, 14% for two applications, 9% for over three applications, and 7% for three applications. This failure rate is significant and the underlying reasons must be identified. Our findings are in line with the results of the Executive Unit for the Financing of Higher Education, Research, Development and Innovation (UEFISCDI) which showed in recent reports regarding the research projects funding competitions that most research proposals will fail to secure funding for their authors: for example, it reported that for the national competitions only 15% from projects were funded in 2020–2021. In the case of Exploratory Research Projects (PCE 2020) the success rate was 24.2%, for Postdoctoral Research Projects (PD 2019) the average success rate was 43.4% while for Research Projects for the stimulation of young independent teams (TE 2019) the average success rate was only 18.5% [28].

We also decided, for testing the hypothesis formulated, to analyse the submissions and success rates of funding applications broken down by gender and fundamental research areas of interest. The results obtained highlight a series of differences.

Therefore, from Table 2 we notice that the number of funding competitions to which the respondents applied differs depending on the main field of research. Thus, most respondents in the field of Social and Economic Sciences applied only to 1 or 2 funding competitions. The results are similar for researchers in the Humanities. While the largest share of researchers belonging to the Natural, Exact and Engineering Sciences applied for 3 or 4 funding competitions.

**Table 2:** Number of competitions in which respondents participated in the last 5 years by respondent’s fundamental area of interest and by their gender.

	Social and economic sciences	Humanities	Natural, Exact and Engineering Sciences	Females	Males
None	25.0%	21.0%	18.1%	24.4%	17.2%
1–2 competitions	**39.7%**	**38.3%**	25.5%	**37.8%**	**29.3%**
3–4 competitions	13.2%	28.4%	**29.8%**	23.0%	25.3%
5–6 competitions	5.9%	6.2%	10.6%	8.2%	8.1%
above 6 competitions	16.2%	6.2%	16.0%	6.7%	20.2%

Source: authors own calculations.

The percentage of those who applied to most competitions is the highest for the Social and Economic Sciences at a short distance from those researchers from Natural, Exact and Engineering Sciences. The Economic and Social Sciences dominate the ranking even when we analyse the percentage of those who did not apply to any competition for funding research projects, with a quarter of respondents. These findings highlight that the field of research plays an important role in the decision of researchers to apply to a competition for research project funding, thus confirming hypothesis 2 formulated above. This can be explained by the eligibility criteria which are different depending on the field of research but also by the publication possibilities specific to the field which favours or limits the fulfilment of these criteria. Therefore, a part of our first hypothesis is confirmed.

Likewise, we found some important differences in terms of participation in research project funding competitions depending on the gender of researchers. Thus, the largest share of respondents for both men and women applied to 1 or 2 competitions. But, going further, we see that the bottom is dominated by a greater proportion of women.

Thus, more women than men did not apply to any research project funding competition. The higher shares for women are maintained when analysing the respondents who participated in 1 or 2 funding competitions. However, men applied in a higher proportion to 3 or 4 financing competitions. For those who applied to 5 or 6 competitions, the percentages are close, slightly higher for women. But, in the case of those who applied to over 6 competitions, the difference is significant in favour of men. These results confirm a part of our third hypothesis, and are in line with the results of other studies in the literature [15, 23, 24, 29, 30] that also point out that women apply less than men in research funding competitions.

Statistics from the Executive Unit for the Financing of Higher Education, Research, Development and Innovation (UEFISCDI) in Romania for the latest research funding competitions show that as the complexity of research projects increases the participation of women researchers in these competitions decreases and increases the participation of men. For the Postdoctoral projects, the project director has the title of doctor obtained no more than 6 years ago, compared to the time of submitting the project proposal. For Young teams projects, the project director has the title of doctor obtained no more than 12 years ago, compared to the time of submitting the project proposal. Thus, if for the post-doctoral projects and young teams projects there are more women who have submitted applications, in the case of exploratory research projects there are more men who have submitted [31]. As in the UEFISCDI report for the 2020 competitions, for Exploratory Research Projects there were submitted: 460 proposals by women and 593 by men, from which 104 were won by women and 143 by men [32].

From Table 3 we observe that the didactic or research rank has a significant influence on the number of competitions in which the respondents participated in the last 5 years.

**Table 3:** Number of competitions in which respondents participated in the last 5 years by didactic or research rank of the respondents (the didactic and research ranks considered are specific to Romania, because there is no ranking system worldwide recognized).

	Research Assistant	Scientific Researcher/Teaching assistant	Scientific Researcher III/University lecturer	Scientific Researcher II/Associate professor	Scientific researcher I/Professor
None	**58.8%**	19.1%	25.0%	13.9%	5.9%
1–2 competitions	17.7%	**61.9%**	**33.7%**	29.2%	**20.6%**
3–4 competitions	17.7%	9.5%	22.8%	**31.9%**	16.2%
5–6 competitions	0. 0%	4.8%	5.4%	13.9%	4.4%
above 6 competitions	5.9%	4.8%	13.0%	11.1%	13.2%

Source: authors own calculations.

Thereby, more than half of those who are research assistants did not participate in any funding competition. This can be justified by their limited experience, the non-fulfilment of the eligibility criteria, which from year to year become more and more severe, making it difficult for researchers at the beginning of their academic road. As they advance in their career, there is a slight increase of the submitted applications followed by a stagnation. Thus, 61% of Scientific Researcher/Teaching assistants applied to 1–2 funding competitions, while 31% of Scientific Researcher II/Associate professors applied to 3–4 funding competitions. Most of the Scientific Researcher III/University lecturer (i.e. 33%) and 20% of Scientific Researcher I/Professor applied for 1–2 funding competitions. These findings confirm a part of our second hypothesis.

When analysing the success rate of the submitted applications (see Table 4), we notice that the Natural, Exact and Engineering sciences field is the one with the highest rejection rate, with a small difference compared to Socio-Economic Sciences and Humanities.

**Table 4:** Number of applications that obtained funding by the fundamental area of interest and by gender.

	Social and economic sciences	Humanities	Natural, Exact and Engineering Sciences	Females	Males
None	**44.9%**	**44.4%**	**46.8%**	**48.2%**	40.4%
1	24.3%	25.9%	22.3%	**25.2%**	22.2%
2	14.4%	17.3%	13.8%	14.1%	**15.2%**
3	7.0%	7.4%	5.3%	4.4%	**10.1%**
above 3	9.5%	4.9%	11.7%	8.2%	**12.1%**

Source: authors own calculations.

But this is also the field with the highest share of applicants who have obtained funding for over 3 projects. The applicants from the Humanities field predominate as a share for one, two or 3 funded applications. Starting from those results obtained above we notice that hypothesis one is confirmed.

Our findings are correlated with the reports of UEFISCDI which also show different success rates depending on the field of interest. For example, in the case of Exploratory Research Projects (PCE 2020) the highest success rate was obtained for the field of Materials Sciences (24.81%) and the lowest rate was obtained for the field of Earth Sciences (23.6%) [32]. For Postdoctoral Research Projects (PD 2019) the highest success rate was obtained for the field of Exact Sciences (Mathematics and Chemistry– 44.4%) while the lowest success rate was obtained for the Economic Sciences (33.3%). In the case of Research Projects for the stimulation of young independent teams (TE 2019) the highest success rate was obtained also for the field of Exact Sciences (Mathematics– 0.8%) while the lowest success rate was obtained for Engineering Sciences (17.7%) [28].

Also, our analysis depicts that, in general, male respondents had more success compared to female ones. Almost half of the women who answered the questionnaire did not receive funding for any project they submitted in grant competitions. On the other hand, only 40% of men did not obtain funding. Regarding respondents who received funding for 3 or more research projects, men are the ones who have higher weights. Thus we notice that the applying men are more likely to get financing for the submitted projects compared to women applicants. These results confirm the third hypothesis and are in accordance with those obtained by other studies [20, 25, 33, 34]. In all these studies the analysis suggested higher rejection rate for female relative to male [20], and the gaps are partly or wholly driven by women being assessed less favourably as principal investigators compared with their male colleagues [34].

In terms of teaching/research ranks, the results show that 82% of the research assistants did not receive funding for any submitted project applications, while only 47% of the Scientific Researcher/Teaching assistants were in a similar situation. Although we would expect the rates to continue the downward trend, 55% of Scientific Researcher III/University lecturers did not obtain funding for any submitted projects. This result can also be correlated with the structure of the sample that holds the largest share of respondents with this teaching or research rank. Going further, 31% of Scientific Researcher II/Associate Professor did not obtain funding for any submitted project, and 36% of Scientific researcher I/Professor obtained funding for a submitted project (see Table 5). Analysing those who obtained funding for over 3 of the submitted projects, we notice that Scientific researcher I/Professor has the highest share, here the ascending trend is obvious as the teaching/research rank increases. Thus, we observe that hypothesis two is confirmed.

**Table 5:** Number of applications that obtained funding by didactic or research rank of the respondents.

	Research Assistant	Scientific Researcher/Teaching assistant	Scientific Researcher III/University lecturer	Scientific Researcher II/Associate professor	Scientific researcher I/Professor
None	**82.4%**	**47.6%**	**55.4%**	**31.9%**	26.9%
1	11.8%	28.6%	16.3%	29.2%	**36.6%**
2	0.0%	14.3%	13.0%	18.1%	17.1%
3	0.0%	4.8%	6.5%	9.7%	7.3%
above 3	5.9%	4.8%	8.7%	11.1%	12.2%

Source: authors own calculations.

For analysing the results of our econometric analysis, we first run the descriptive statistics for the variables considered. The independent variables included take different values. Main field of research takes the value 1 for Social and Economic sciences, the value 2 for Humanities and the value 3 for Natural, Exact and Engineering Sciences. The Specific field variable takes value from 1 to 13 describing each specific field considered. The didactic or research rank takes values between 1 and 5, starting from the lower didactic or research ranks to the highest ones. Table 6 shows the minimum, and maximum values but also the mean and standard deviation for all the variables included in the analysis. We can observe that the mean for Participation is 0.79 which indicates that, on average 79% of the respondents chose the answer”yes” showing their participation in research funding competitions in the last five years. As regards success rates the mean value shows that only 56% from the applicants obtained funding in research funding competitions in the last five years. The mean value for the main field is showing that the respondents are somehow evenly distributed between domains. On the other hand, the average value obtained for the Specific Field variable shows a distribution of respondents to the right, to the higher values of this variable, for example, a higher share of respondents come from the fields of social sciences and humanities. This result is also confirmed by the graphical representation in Fig 1.

**Table 6:** Descriptive statistics of the variables included in the logistic model estimation.

	Minimum	Maximum	Mean	Standard Deviation
Participation	0.0	1.0	0.8	0.41
Success	0.0	1.0	0.6	0.50
Main field	1.0	3.0	2.1	0.81
Specific field	1.0	13.0	8.9	2.74
Academic rank	1.0	5.0	3.4	1.09
Gender	0.0	1.0	0.6	0.50

Source: authors own calculations.

The mean value for the didactic or research rank shows that in the sample we have more respondents who have higher teaching or research ranks.

Table 7 summarizes the results of the logistic model estimation. The results for Model 1 revealed that the Main field, Academic rank and Age are significant determinants of the participation of researchers in research funding competitions. Therefore, the participation in research funding competitions is positively related to the Main field of interest and the professional level experience of the applicants. This shows that researchers belonging to the field of Natural, Exact and Engineering Sciences have higher participation rates to research funding competitions, compared to those in the field of Humanities and Social and Economic Sciences.

**Table 7:** Logistic model estimation results.

Model	Model 1			Model 2		
Dependent variables	Participation rate			Success rate		
Independent variables	Coefficient B (S.E.)	Exp (B)	Wald	Coefficient B (S.E.)	Exp (B)	Wald
Constant	1.19 (1.32)	3.29	0.83	-0.32 (1.15)	0.73	0.08
Main field	0.42* (0.24)	**1.52**	**3.03**	0.05 (0.19)	1.05	0.07
Specific field	0.08 (0.07)	1.08	1.22	0.05 (0.06)	1.05	0.63
Academic rank	0.75*** (0.28)	**2.11**	**6.95**	0.98*** (0.25)	**2.66**	**14.81**
Gender	-0.22 (0.37)	0.81	0.34	-0.12 (0.30)	0.89	0.15
Age	-0.11*** (0.03)	**2.40**	**11.36**	-0.07** (0.03)	**0.94**	**4.86**
Chi-square	39.86***			41.33***		
R square	0.24			0.22		

Note: *, ** and *** represents statistically significant at 10%, 5% and 1% respectively.

Source: processed by the authors.

Participation in research funding competitions is also positively related with the professional level experience measured by the didactic or research rank, showing that as researchers have higher experience their rate of participation in competitions is higher. This increase in the participation rate can be based on two reasons: (1) applicants are motivated by the intention to be promoted to higher professional ranks, and (2) the capacity to raise funds is becoming a needed skill in managing research groups/ laboratories.

On the other hand, the participation in research funding competitions is negatively related with the age of the researchers, showing that younger researchers are more interested in participating in research funding competitions. Thus, younger researchers with higher teaching or research ranks are more interested and submit more proposal projects in research funding competitions. These results confirm hypotheses 1 and 2 previously formulated, but reject hypothesis 3. Because, from an econometric point of view, gender did not result in having a statistically significant influence on the participation in research funding competitions.

The results of Model 2 emphasize that Academic rank and Age are significant determinants of the success rates in research funding competitions. Thus, younger researchers with higher teaching or research ranks are more interested and submit more projects in research funding competitions. These results confirm hypothesis 5 and reject hypotheses 4 and 6 because the main field and gender did not result in having a statistically significant influence on the success rate of the respondents in research funding competitions.

## Conclusions

The main purpose of our paper was to identify the determinant factors of the participation and success rates of the researcher in research funding competitions, in the case of Romania. To achieve this purpose, we used different methods of analysis.

The main findings of the empirical analysis emphasize that the participation of the researchers from Romanian universities in research funding competitions is influenced by their main field of interest and professional level experience. Age and gender are also significant determinants of participation in research funding competitions. Thus, we found that men participated in more competitions compared to women. Also, younger researchers with higher teaching or research ranks participated to a greater extent in these competitions. Those who are interested in the field of exact sciences have had higher levels of participation compared to other fields of research (such as social and humanities sciences).

In terms of success rates, our results show that, for Romanian researchers, the success in grant competitions depends on their professional level experience, age and gender. Therefore, the researchers with the highest success rates are men, but also younger researchers with higher teaching or research ranks.

Our results complement the findings in the literature which consider gender as a determinant of participation and success rates in research funding competitions. Thus, the results show that men participate more in such competitions and have higher success rates compared to women. The novelty part, which differentiates our research from other studies is the fact that we emphasize that young researchers with higher teaching or research ranks have both higher participation rates and increased success rates in competitions for funding their research.

The study has some limitations which come from the limited number of participants who answered the questionnaire. For the further development of this topic, we intend to extend the questionnaire in order to identify the main reasons why the projects received or did not fund in the research funding competitions by analysing the reasons from the opinion of the researchers but also of the evaluators. We also intend to apply the questionnaire to other countries in Central and Eastern Europe in order to make a comparison with the case of Romania and identify the patterns that characterize grant competitions in science. Also, the quantitative analysis should be corroborated with a qualitative approach in order to complete the successful applicant profiles.

## Supporting information

S1 Data (ZIP)
